# Temporal Trends of Severe Hypoglycemia and Subsequent Mortality in Patients with Advanced Diabetic Kidney Diseases Transitioning to Dialysis

**DOI:** 10.3390/jcm8040420

**Published:** 2019-03-27

**Authors:** Ching-Chung Hsiao, Hui-Tzu Tu, Chi-Hung Lin, Kuan-Hsing Chen, Yung-Hsin Yeh, Lai-Chu See

**Affiliations:** 1 Kidney Research Center and Department of Nephrology, Chang Gung Memorial Hospital, Taipei 333, Taiwan; colinhua0123@gmail.com (C.-C.H.); guanhsing3795@gmail.com (K.-H.C.); 2 Graduate Institute of Clinical Medical Sciences, Chang Gung University, Taoyuan 333, Taiwan; 3 Department of Public Health, College of Medicine, Chang Gung University, Taoyuan 333, Taiwan; tzujob@gmail.com; 4 Center for Big Data Analytics and Statistics, Chang Gung Memorial Hospital at Linkou, Taoyuan 333, Taiwan; 5 Center for Artificial Intelligence in Medicine, Chang Gung Memorial Hospital at Linkou, Taoyuan 333, Taiwan; lin3031@gmail.com; 6 Division of Rheumatology, Allergy and Immunology, Department of Internal Medicine, Chang Gung Memorial Hospital, Linkou, Taoyuan 333, Taiwan; 7 The Cardiovascular Department, Chang Gung Memorial Hospital at Linkou, Taoyuan 333, Taiwan; yeongshinn@cgmh.org.tw; 8 College of Medicine, Chang Gung University, Taoyuan 333, Taiwan; 9 Biostatistics Core Laboratory, Molecular Medicine Research Center, Chang Gung University, Taoyuan 333, Taiwan

**Keywords:** advanced diabetic kidney disease, severe hypoglycemia, dialysis

## Abstract

Background: Patients with diabetic kidney disease (DKD) are at higher risk of hypoglycemia than diabetic patients without DKD. We aimed to investigate the temporal trends of severe hypoglycemia in advanced DKD patients transitioning to dialysis and examine risk factors associated with severe hypoglycemia. We also investigated the association of severe hypoglycemia episodes with one-year mortality after initiation of dialysis in patients with advanced DKD. Methods: Using the Taiwan National Health Insurance Research Database, 46,779 advanced DKD patients transitioning to dialysis (Peritoneal dialysis 4216, hemodialysis 42,563) between 1997 and 2011 were enrolled. We calculated the rates of severe hypoglycemia from 5 years before dialysis until 10 years after dialysis. Cox proportional hazard model was used to examine the risk factors of post end stage renal disease (ESRD) one-year hypoglycemia and post ESRD one-year mortality in advanced DKD patients transitioning to dialysis. Results: We found that 11.5% of advanced DKD patients had at least one episode of severe hypoglycemia the year leading up to dialysis initiation. Multivariate analysis revealed hemodialysis compared with peritoneal dialysis, stroke, use of sulfonylurea, glinide, and insulin were associated with higher risk of severe hypoglycemia one year after transitioning to dialysis. Increased frequency of severe hypoglycemia-related hospitalizations was associated with incrementally higher mortality risk one year after transitioning to dialysis (Pre-ESRD hypoglycemia: Hazard ratios: 1.28 (1.18–1.38, *p* < 0.001), 1.64 (1.49–1.81, *p* < 0.001) for one, two hypoglycemia-related hospitalizations, respectively; post-ESRD hypoglycemia: HRs of 1.56 (1.40–1.73, *p* < 0.001), 1.72 (1.39–2.12, *p* < 0.001) for one, two hypoglycemia-related hospitalizations, respectively (reference group: no hypoglycemia related hospitalization)). Conclusions: Among advanced DKD patients, we observed a progressive elevated risk of hypoglycemia during the critical dialysis transition period. Increased frequency of severe hypoglycemia-related hospitalizations was associated with higher mortality risk one year after transitioning to dialysis. Further study of glycemic management strategies which prevent hypoglycemia during the critical transition period are warranted.

## 1. Introduction

Diabetes mellitus (DM) is the most common cause of chronic kidney disease (CKD) and end stage renal disease (ESRD) in many countries throughout the world [1]. Rigorous glucose control is a key goal to prevent ESRD in patients with diabetic kidney disease (DKD). However, in the ADVANCE and the ACCORD studies, intensive blood glucose lowering was associated with an increased risk of severe hypoglycemia [2,3]. Furthermore, loss of kidney function interferes with glucose homeostasis and confound glycemic control. Advanced DKD patients progressing to dialysis may be susceptible to hypoglycemia due to decreased gluconeogenesis in the diseased kidneys, malnutrition due to uremia, altered oral hypoglycemic agents and insulin metabolism, and comorbidities [4,5].

According to the 2017 United States Renal Data system (USRDS) report, patients with DM appeared to exhibit a gradual fall in serum glucose levels over time, as their CKD progressed to ESRD. Advanced DKD is not only a risk for hypoglycemia, but also increases the severity of hypoglycemia [6]. After dialysis initiation, Kalantar-Zadeh et al demonstrated that one-third of diabetic hemodialysis patients may experience spontaneous normalization of hyperglycemia and frequent hypoglycemic events, a phenomenon termed “burnt-out diabetics” [7].

Mild degree of hypoglycemia may cause dizziness, disorientation, or slurred speech, while severe hypoglycemia, which required medical assistance, may present with seizure, coma, cardiac ischemia, arrhythmias, or sudden death [8]. The initiation of dialysis has pronounced effects on the physiology of the patient; however, few studies have evaluated the impact of such changes on the occurrence of severe hypoglycemia, which required emergency department (ED) visits or hospitalizations during the critical transition period of dialysis initiation, including the pre-dialysis period in patients with advanced DKD. Hence, to address this knowledge gap, we investigated the temporal trends of severe hypoglycemia in advanced DKD patients transitioning to dialysis. Regarding the impact of different dialysis modality, we also compared the severe hypoglycemia risks between hemodialysis and peritoneal dialysis. To further survey modifiable risk factors of severe hypoglycemia in the dialysis transition period, Cox proportional hazard models were performed to evaluate risk factors associated with post-ESRD one-year severe hypoglycemia.

Finally, owing to high mortality risks being observed particularly in the period of advanced DKD transitioning to dialysis [9,10], we sought to determine pre-ESRD severe hypoglycemia episodes (emergency department visits or hospitalizations) or post-ESRD one year severe hypoglycemia episodes (emergency department visits or hospitalizations) were associated with post ESRD one-year mortality.

## 2. Materials and Methods

### 2.1. Data Source

We conducted a retrospective cohort study with longitudinal data from the Taiwan National Health Insurance Research Database (NHIRD). The Taiwan National Health Insurance (NHI) program is a nationwide, compulsory health care program covering approximately 99.9% of Taiwan’s population, which stood at approximately 23.37 million in 2014. Before 2000, diagnoses in claims data were coded using A codes, followed by International Classification of Diseases, Ninth Revision, Clinical Modification (ICD-9-CM) codes after 2000. The NHIRD contains the comprehensive health care information of insured patients, including disease diagnoses, inpatient orders, outpatient visits, drug prescriptions, and registries of beneficiaries with specific conditions, but it does not include laboratory data. The requirement to obtain informed consent was waived because data in the NHIRD that could identify specific patients is scrambled and encrypted before being released to researchers. Consistent data encryption made linking and continuously following all claims belonging to the same patient within the NHIRD feasible. The study protocol was approved by the Institutional Review Board, Chang Gung Medical Foundation, Taiwan (IRB No: 201702335B1).

In the NHI program, patients with some specific chronic conditions, including ESRD, malignancies, qualify for a catastrophic illness certificate. To qualify for a certificate, a patient’s condition must be repeatedly verified by a peer review group based on clinical evidence, pathologic findings, and laboratory data. The Registry for Catastrophic Illness Patient Database (RCIPD) is an NHIRD subset that comprises the data of patients with a certificate, including those with ESRD and permanent dialysis.

### 2.2. Patient Selection and Definition of Dialysis Modality

**Figure 1:**
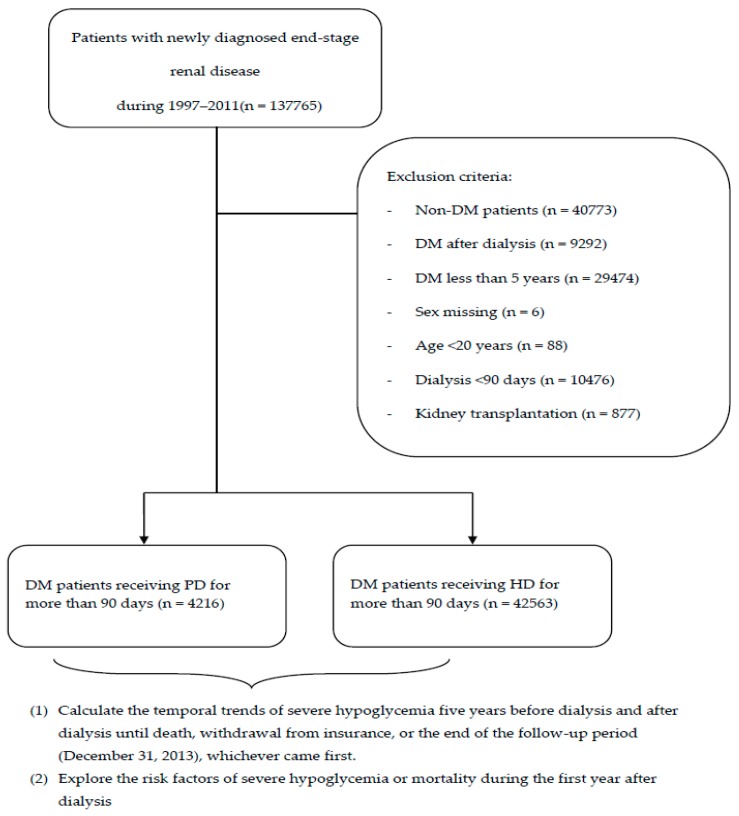
Flow chart demonstrating enrollemnt of the study cohort. (DM: diabetic mellitus; PD: peritoneal dialysis; HD: hemodialysis.).

Figure 1 demonstrates the process used for selecting the participants in the study cohorts. Patients who were newly diagnosed with ESRD (ICD-9-CM code: 585; A code: A350) from 1 January 1997 to 31 December 2011 were identified from the Taiwan NHIRD. Some peritoneal dialysis (PD) patients temporarily receive hemodialysis (HD) at the initiation of dialysis; therefore, dialysis modality was defined as the modality at day 90 after the first dialysis session. Because some patients suffering from acute kidney injury lead to temporary dialysis, patients who underwent dialysis for less than 90 days were excluded (*n* = 10,476). Index date was defined as the date of starting the first dialysis. Patients with DM were defined by at least three outpatient claims or one inpatient claims with ICD-9-CM code = 250. We exclude patients diagnosed DM in less than five years (*n* = 29,474) because we calculated the 5-year rate of severe hypoglycemia before dialysis. Patients without DM (*n* = 40,773), diagnosed with DM after dialysis (*n* = 9292), <20 years of age (*n* = 88), with missing sex information (*n* = 6), and who subsequently received kidney transplantation (ICD-9-CM: V42.0) after dialysis (*n* = 877) were excluded. Finally, 42,563 diabetic patients undergoing HD and 4216 diabetic patients undergoing PD were enrolled in the study cohort.

### 2.3. Outcome Variables

The outcomes of primary interest were the occurrence of severe hypoglycemia during the course of CKD progression until 10 years after dialysis initiation.

Severe hypoglycemia was defined as patients who visited the emergency department (ED) with a diagnosis of hypoglycemia or a primary discharge diagnosis of the following ICD-9-CM codes: 251.0 (hypoglycemic coma), 251.2 (unspecified hypoglycemia), and 251.8 (diabetic hypoglycemia; hypoglycemic shock) in the inpatient database.

The secondary outcomes were post-ESRD one-year severe hypoglycemia and post-ESRD one-year mortality. Mortality was defined by withdrawal from the NHI program or death in NHIRD or RCIPD. All participants were followed from the index date until the first occurrence of hypoglycemia, death, withdrawal from insurance, or the end of the follow-up period (31 December 2013), whichever came first.

### 2.4. Baseline Characteristics

The covariates were age, sex, comorbidities, initial dialysis type, and medications.

Comorbidities were identified when reported for more than 2 outpatient visits or one inpatient stay within the previous year of index date. Comorbidities included coronary artery disease (ICD-9-CM codes 410–413, 414.01–414.05, 414.8, and 414.9; A codes A270 and A279); congestive heart failure (ICD-9-CM codes 428, 398.91, and 402.x1; A codes A251, A260, and A289); stroke (ICD-9-CM codes 430–438; A codes A290, A291, A292, and A299); hyperlipidemia (ICD-9-CM code 272; A code A182); atrial fibrillation (ICD-9-CM code 427.31; A code 281); hypertension (ICD-9-CM codes 401 and 402; A code A26); and liver cirrhosis (ICD-9-CM code 571.5; A code A347). Most diagnostic codes used for these comorbidities were validated in previous NHIRD-based studies [11,12]. Medications were identified by the filling of a prescription at least twice or refilling a prescription for a chronic illness at least once in the previous 3 months of the index date. Medication included sulfonylurea, glinides, thiazolidinediones (TZD), acarbose, dipeptidyl peptidase 4 inhibitors (DPP4 inhibitors), and insulin.

### 2.5. Statistical Analysis

The rates of severe hypoglycemia 5 years before dialysis until 10 years after dialysis were calculated in advanced DKD patients. The rates between dialysis modalities were compared using inverse probability of treatment weighting (IPTW), by calculating the propensity scores based on baseline variables, which were used to balance the differences between the PD and HD patients. Logistic regression was used to calculate the propensity score for each patient by estimating the probability of assignments based on baseline variables, including age, sex, comorbidity, and medication. The balance of covariates at baseline between the two dialysis groups was assessed using absolute standardized mean difference (ASMD), which is not influenced by sample size. An ASMD value of ≤0.1 indicates a negligible difference in potential confounders between the two dialysis groups [13]. Survival analyses (log-rank test in univariate analysis and Cox’s proportional hazard model analysis) were conducted to determine the effects of covariates on hypoglycemia. The unadjusted and adjusted hazard ratio (HR and aHR) and its 95% confidence interval (CI) were calculated to demonstrate the relative risk of severe hypoglycemia for the covariates of interest. Survival analysis without propensity score weighting was conducted to evaluate the effects of covariates on hypoglycemia. For one-year mortality after dialysis, survival analysis was also performed to evaluate the effect of covariates, pre-ESRD severe hypoglycemia, and post-ESRD severe hypoglycemia. All analyses were performed using SAS 9.4 (SAS Institute, Cary, NC, USA); a *p* value of 0.05 was considered statistically significant.

## 3. Results

### 3.1. Subject Characteristics

Table 1 presents the baseline characteristics of the HD cohort when compared with the PD cohort before and after IPTW. The mean age (years) was 65.83 ± 11.55 in the HD cohort and 60.38 ± 12.89 in the PD cohort. The proportion of patients with stroke, coronary artery disease, and congestive heart failure, as well as the prescription rates of sulfonylurea and insulin, were significantly higher in the HD cohort than in the PD cohort. After propensity score weighting, baseline characteristics were balanced between the two cohorts.

### 3.2. Temporal Trends of Hypoglycemia in Advanced DKD Patients Transitioning to Dialysis

**Figure 2:**
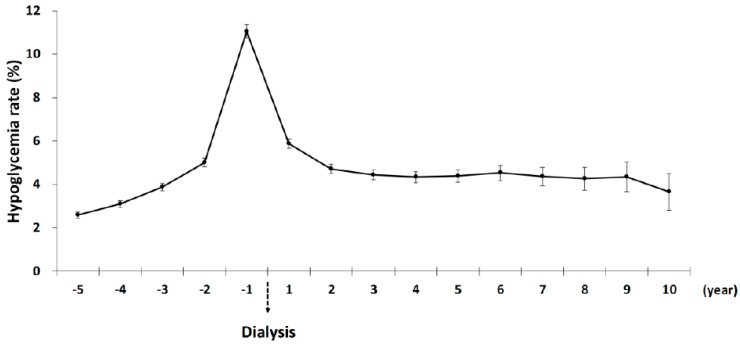
Temporal trends of severe hypoglycemic rates in diabetic kidney patients before and after dialysis.

Figure 2 shows the temporal trends of severe hypoglycemia in patients with advanced DKD transitioning to dialysis. A gradual increase in rates of severe hypoglycemia occurred as time advanced before the initiation of dialysis. A total of 11.5% of Advanced DKD patients had at least one episode of severe hypoglycemia the year leading up to dialysis initiation. One year after the start of dialysis, the rate of severe hypoglycemia remained high (5.88%), but declined compared with the pre-dialysis period. Two years after dialysis, the hypoglycemic rates were stable at around 4%.

### 3.3. Comparison of Hypoglycemic Rates between the HD and PD Cohorts

**Figure 3:**
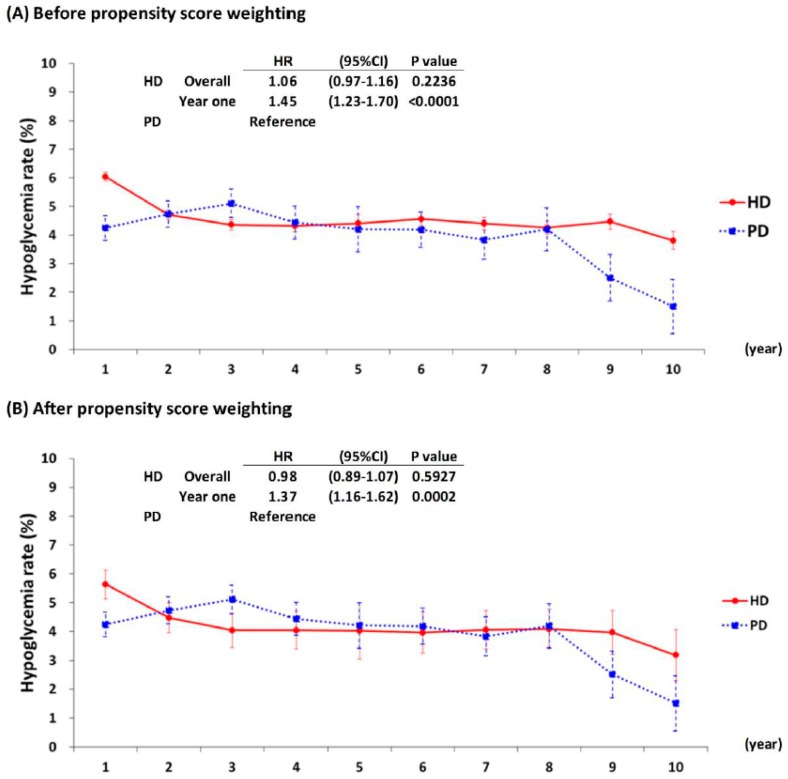
Severe hypoglycemic rates after dialysis among diabetic kidney patients.

Figure 3 presents the rates of severe hypoglycemia in the HD cohort compared with the PD cohort. The overall hypoglycemic risk in the HD cohort were not significantly different from those of the PD cohort. However, the risk in the first year after dialysis initiation were significantly higher in the HD cohort compared with the PD cohort before propensity score weighting (HR = 1.45 (1.23–1.7); *p* < 0.001) and after (aHR = 1.37 (1.16–1.62); *p* < 0.001) propensity score weighting.

### 3.4. Risk Factors of One-Year Severe Hypoglycemia among Advanced DKD Patients after Transitioning to Dialysis

Table 2 presents the risk factors of severe hypoglycemia in advanced DKD patients one year after transitioning to dialysis. The Cox’s proportional hazard model revealed hemodialysis compared with peritoneal dialysis (aHR = 1.29; 1.12–1.30; *p* < 0.001) and stroke (aHR = 1.16; 1.07–1.25; *p* < 0.001) were associated with severe hypoglycemia one year after dialysis. Use of antidiabetic agents, especially sulfonylurea (aHR = 1.31; 1.20–1.44; *p* < 0.001), glinide (aHR =1.20; 1.1–1.30; *p* < 0.001), and insulin (aHR = 2.63; 2.32–2.98; *p* < 0.001), predisposed patients to higher risks of severe hypoglycemia one year after dialysis. By contrast, patients with hypertension or hyperlipidemia were associated with lower risks of severe hypoglycemia one year after dialysis.

### 3.5. Risk Factors of One-Year Mortality among DKD Patients Transitioning to Dialysis

Table 3 presents the risk factors of one-year mortality among DKD patients transitioning to dialysis. Pre-dialysis characteristics, old age (Age > 65), stroke, heart failure, liver cirrhosis, and pre- or post-ESRD severe hypoglycemia episodes were positively associated with one-year mortality. Frequency of hypoglycemia-related hospitalizations was associated with higher mortality risk compared to no hypoglycemia-related hospitalizations (Pre-ESRD hypoglycemia: 1 versus none, aHR = 1.28, 1.18–1.38; *p* < 0.001; 2 versus none, aHR = 1.64, 1.49–1.81; *p* <0.001; Post-ESRD hypoglycemia; 1 versus none, aHR = 1.56, 1.40–1.73; *p* < 0.001; 2 versus none, aHR = 1.72, 1.39–2.12; *p* < 0.001). Hemodialysis compared with peritoneal dialysis was associated with higher one-year mortality (aHR = 1.64, 1.44–1.87; *p* < 0.001). By contrast, hypertension and hyperlipidemia were negatively associated with one-year mortality.

## 4. Discussion

The present study demonstrated that the risk of severe hypoglycemia gradually increased in patients with advanced DKD transitioning to dialysis. We found that 11.5% of advanced DKD patients had at least one episode of severe hypoglycemia the year leading up to dialysis initiation. Hemodialysis compared with peritoneal dialysis, stroke, use of insulin, sulfonylurea, and glinide were associated with higher risk of severe hypoglycemia in the critical transition period. We observed a dose response relationship between increased frequency of severe hypoglycemia-related hospitalizations and incrementally higher one-year mortality, regardless if its occurrence was before or after dialysis.

### 4.1. Trajectory of Severe Hypoglycemia over the Dialysis Transition Period

Our study describes the trajectory of severe hypoglycemia over the dialysis transition period and demonstrated a gradual increase in severe hypoglycemia as time advanced before dialysis initiation. This may be explained by the differences in glucose metabolism among DKD patients and DM patients without kidney disease. Renal clearance of insulin and degradation of insulin in peripheral tissues are decreased in patients with CKD [14,15]. With the progressive decline in renal mass, decreased renal gluconeogenesis frequently occurs in these patients [14]. Moreover, most antidiabetic drugs have a prolonged half-life in patients with CKD because these drugs are eliminated through renal excretion. In secondary analysis of the ACCORD study, patients with mild to moderate CKD (stage 1–3) were associated with nearly a two-fold higher risk of hypoglycemia compared with those of normal renal function [16]. In another large population-based study involving 243,222 veterans, the incidence of hypoglycemia, which was defined by blood glucose level <70 mg/dL, was higher in patients with CKD versus without CKD [6]. However, the above studies mainly focus on mild to moderate CKD and did not demonstrate the different hypoglycemia risk as CKD progression. Our study gives an insight into the heightened risk of severe hypoglycemia in patients with advanced DKD during the dialysis transition period (one year before and after dialysis). Patients with advanced DKD may present with symptoms of uremia, such as decreased oral intake, nausea, and vomiting, leading to malnutrition and increased hypoglycemia [17]. The constellations of the aforementioned factors predispose patients with advanced DKD to higher risks of hypoglycemia. Thus, a spontaneous decrease in hemoglobin A1c levels in the absence of any treatment in the period of advanced CKD transitioning to dialysis may be considered a “hypoglycemia alarm”, rather than true improvement by the primary care physician. Without medication adjustments, patients may encounter hypoglycemia leading to hospitalizations.

This large population-based study used propensity score weighting to compare risk of severe hypoglycemia between HD and PD patients. This method reduced the unbalance inherent to retrospective cohort studies. In this study, similar risks of developing severe hypoglycemia were observed between the HD and PD patients, except high risk in the HD cohort than in the PD cohort during the first year of dialysis. Several studies have reported higher risks of mortality and stroke during the first year following the start of HD [18,19,20]. Pathophysiological changes such as rapid toxin and fluid removal, infection related to central venous catheter, inflammation related to repeated needle puncture, and rapid decline of residual renal function are more pronounced after HD initiation. Whether the dialysis treatment itself, the progression of comorbid diseases, or the rapid decline of residual renal function accounted for the increased risk of severe hypoglycemia during the first year of HD is uncertain and warrants further investigation. Nevertheless, our study provides insight into the risk of severe hypoglycemia, which required medical attention during dialysis transition period, especially when a patient chooses hemodialysis as modality.

Risk factor analyses of one-year severe hypoglycemia after transitioning to dialysis revealed that hemodialysis compared with peritoneal dialysis and stroke were associated with higher risk of severe hypoglycemia, whereas hypertension and hyperlipidemia were associated with lower risk of severe hypoglycemia in advanced DKD patients transitioning to dialysis. Borzi et al. reported that hypoglycemia was more frequent among patients with cognitive dysfunction [21]. Stroke may lead to an increased risk of hypoglycemia because of impaired cognitive function, difficulty in ambulation, and poor nutrition. With regard to the various antidiabetic agents used in advanced DKD patients, sulfonylurea, glinide, and insulin were found to be associated with higher risks of hypoglycemia in the present study. Previous studies have demonstrated that insulin requirements decrease by 25% when the GFR falls below 50 mL/min/1.73 m^2^, and by a further 50% when it falls below 10 mL/min/1.73 m^2^ [15,22,23]. Renal clearance of insulin and insulin catabolism in peripheral tissues is also decreased in patients with advanced DKD [14]. The glucose-lowering effect of insulin persists for a long duration and increases the risk of symptomatic hypoglycemia. After the initiation of dialysis, increased insulin resistance in the uremic milieu improves and further decreases insulin requirements [24]. The metabolism of sulfonylurea, which belongs to a class of long-acting antidiabetic agents, is prolonged in patients with advanced DKD. Previously, two studies have demonstrated that patients with CKD experienced severe hypoglycemia, which required ED visits or hospitalization, especially in patients who received insulin and sulfonylurea treatments [25,26]. Thus, monitoring the trend of the glucose level and dosing adjustments of sulfonylurea, glinide, and insulin to prevent hypoglycemia are particularly crucial and may decrease the mortality risks in advanced DKD patients transitioning to dialysis.

### 4.2. One-Year Mortality after Dialysis 

High mortality risks were observed particularly in the period of non-dialysis dependent (NDD) advanced DKD transitioning to dialysis dependent ESRD [9,10]. Our study gave an insight into the long-term sequelae of severe hypoglycemia on mortality risks of advanced DKD transitioning to dialysis, and demonstrated increased frequency of severe hypoglycemia-related hospitalizations were associated with higher risks of one-year mortality. In a secondary analysis of the ACCORD trial, intensive glycemic control was associated with a 31% higher risk all cause mortality compared with standard therapy in patients with CKD, but not in patients without CKD [16]. Though the relationship between mortality and hypoglycemia is not direct examined, the increased mortality may partially be explained by frequent hypoglycemia in patients with CKD during intensive glycemic control. Hypoglycemia-related hospitalizations and CKD were independently associated with higher mortality in 8767 diabetic patients from the Hong Kong Diabetes Registry [27]. Hypoglycemia was reported to be associated with increased one-day mortality in a large population-based study in Veterans [6]. Hypoglycemia is linked to increased short-term mortality, possibly related to encephalopathy, seizure, and coma. The adrenergic surge, in the setting of hypoglycemia, aggravated pre-existing underlying disorders, including cardiac arrhythmia, vasoconstriction, and sudden cardiac death [8]. Regarding long term sequelae, recurrent hypoglycemia events were reported to decrease autonomic system responses and further aggravate cardiovascular risk [28]. Furthermore, frequent severe hypoglycemia may reflect underlying comorbidities including uremia, protein energy malnutrition, bedridden status, which might contribute to higher mortality during the critical dialysis transition period. Our study demonstrated that severe hypoglycemia in the dialysis transition period was not only a sign of poor sugar control but was also associated with death. Further studies are warranted to elicit the mechanism by which severe hypoglycemia contributes to higher mortality in DKD patients in the transition period.

This study had several limitations. First, the NHIRD is based on administrative data, which may be subject to data recording and coding errors. Second, the renal metabolism of antidiabetic agents differs at various stages of CKD. Unfortunately, laboratory data such as creatinine, glucose, and glycated hemoglobin levels were not available in the Taiwan NHIRD. Third, we were unable to access the severity of comorbidities (blood pressure, heart function, and lipid level), which could contribute to the development of hypoglycemia. Unavailability of laboratory data and concomitant disorders severity may limit the generalizability and usefulness of our study. Fourth, only patients with severe hypoglycemia, which requiring an ED visit and hospitalization, were considered in this study to avoid the double counting of hypoglycemic events and improve the accuracy of the outcome. Thus, the reported hypoglycemic rate might be underestimated. Finally, although we selected numerous variables for the propensity score method, residual confounding by unmeasured factors cannot be ruled out.

## 5. Conclusions

Progressive heightened risk of severe hypoglycemia was observed in advanced DKD patients transitioning to dialysis. Severe hypoglycemia episodes were associated with an incremental higher risk of mortality in the transition period. Use of anti-diabetic agents, especially sulfonylurea, glinide, and insulin, warrants close glucose level monitoring and dose adjustments to prevent hypoglycemia. Physicians must pay more attention to severe hypoglycemia, which might prove to be a concern in glycemic management in advanced DKD patients transitioning to dialysis. Further study of glycemic management strategies which prevent severe hypoglycemia during the critical transition period are warranted.

## Figures and Tables

**Table 1:** Baseline characteristics at starting dialysis among diabetic kidney patients undergoing hemodialysis and peritoneal dialysis.

	Before Propensity Score Weighting			After Propensity Score Weighting		
	HD	PD	ASMD	HD	PD	ASMD
	(*n* = 42,563)	(*n* = 4216)		(*n* = 42,563)	(*n* = 4216)	
Age (years)	65.83 ± 11.55	60.38 ± 12.89	0.4452	60.45 ± 3.96	60.38 ± 12.89	0.0072
Male	49.25%	50.62%	0.0274	50.76%	50.62%	0.0029
Stroke	43.93%	33.37%	0.2181	33.53%	33.37%	0.0032
CAD	57.63%	48.58%	0.1821	51.26%	48.58%	0.0033
CHF	61.32%	49.95%	0.2302	50.11%	49.95%	0.0032
Hyperlipidemia	73.73%	75.88%	0.0494	75.85%	75.88%	0.0006
AF	7.77%	6.95%	0.0314	6.98%	6.95%	0.0012
Hypertension	96.97%	95.66%	0.0696	95.69%	95.66%	0.0014
LC	17.57%	14.54%	0.0826	14.56%	14.54%	0.0006
Use of sulfonylurea	62.86%	55.03%	0.1598	55.27%	55.03%	0.0048
Use of TZD	21.45%	22.06%	0.0147	22.19%	22.06%	0.0031
Use of glinide	40.91%	38.14%	0.0567	38.32%	38.14%	0.0037
Use of DPP4	5.52%	6.19%	0.0287	6.2%	6.19%	0.0004
Use of acarbose	27.79%	27.35%	0.0099	27.53%	27.35%	0.0042
Use of insulin	73.9%	68.15%	0.1272	68.29%	68.15%	0.0031

HD, hemodialysis; PD, peritoneal dialysis; ASMD, absolute standardized mean difference; CAD, coronary artery disease; CHF, congestive heart failure; AF, atrial fibrillation; LC, liver cirrhosis; TZD, thiazolidinediones; DPP4, dipeptidyl peptidase 4 inhibitors.

**Table 2:** Risk factors of one-year severe hypoglycemia among advanced diabetic kidney patients transitioning to dialysis.

	One Year					
	Univariate			Multivariate		
	HR	(95% CI)	*p* Value	HR	(95% CI)	*p* Value
Dialysis (HD vs. PD)	1.40	(1.2–1.64)	<0.0001	1.29	(1.12–1.30)	0.0017
Male	0.96	(0.88–1.03)	0.2651	--	--	--
Age (65+ vs. <65)	1.03	(0.95–1.11)	0.4996	--	--	--
Stroke	1.21	(1.12–1.31)	<0.0001	1.16	(1.07–1.25)	0.0003
CAD	1.05	(0.97–1.14)	0.2391	--	--	--
CHF	1.10	(1.01–1.19)	0.0234	--	--	--
Hyperlipidemia	0.91	(0.84–1.00)	0.0415	0.85	(0.78–0.93)	0.0003
AF	1.00	(0.87–1.14)	0.9799	--	--	--
Hypertension	0.81	(0.65–1.02)	0.0666	0.67	(0.54–0.85)	0.0007
LC	1.01	(0.91–1.12)	0.8813	--	--	--
sulfonylurea	1.58	(1.44–1.72)	<0.0001	1.31	(1.20–1.44)	<0.0001
TZD	1.23	(1.12–1.34)	<0.0001	--	--	--
Glinide	1.42	(1.31–1.54)	<0.0001	1.20	(1.10–1.30)	<0.0001
DPP4	1.02	(0.86–1.2)	0.8644	--	--	--
Acarbose	1.26	(1.16–1.37)	<0.0001	--	--	--
Insulin	2.88	(2.55–3.26)	<0.0001	2.63	(2.32–2.98)	<0.0001

HD, hemodialysis; PD, peritoneal dialysis; CAD, coronary artery disease; CHF, congestive heart failure; AF, atrial fibrillation; LC, liver cirrhosis; TZD, thiazolidinediones; DPP4, dipeptidyl peptidase 4 inhibitors; HR, hazard ratio; CI, confidence interval.

**Table 3:** Risk factors of one-year mortality among DKD patients transitioning to dialysis.

	One Year					
	Multivariate Model 1			Multivariate Model 2		
	HR	(95% CI)	*p* Value	HR	(95% CI)	*p* Value
Dialysis (HD vs. PD)	1.64	(1.44–1.87)	<0.0001	1.66	(1.46–1.89)	<0.0001
Male	--	--	--	--	--	--
Age (65+ vs. <65)	2.21	(2.07–2.36)	<0.0001	2.19	(2.05–2.33)	<0.0001
Stroke	1.39	(1.31–1.47)	<0.0001	1.40	(1.32–1.48)	<0.0001
CAD	0.94	(0.88–1.00)	0.0487	--	--	--
CHF	1.16	(1.09–1.24)	<0.0001	1.15	(1.08–1.22)	<0.0001
Hyperlipidemia	0.69	(0.65–0.73)	<0.0001	0.69	(0.65–0.73)	<0.0001
AF	--	--	--	--	--	--
Hypertension	0.56	(0.48–0.65)	<0.0001	0.56	(0.48–0.65)	<0.0001
LC	1.28	(1.20–1.37)	<0.0001	1.28	(1.20–1.37)	<0.0001
Pre-ESRD hypoglycemia episodes						
1 versus none	1.28	(1.18–1.38)	<0.0001			
2 versus none	1.64	(1.49–1.81)	<0.0001			
Post-ESRD hypoglycemia episodes						
1 versus none	1			1.56	(1.40–1.73)	<0.0001
2 versus none				1.72	(1.39–2.12)	<0.0001

DKD, diabetic kidney disease; HD, hemodialysis; PD, peritoneal dialysis; CAD, coronary artery disease; CHF, congestive heart failure; AF, atrial fibrillation; LC, liver cirrhosis.
